# International challenges without borders: a descriptive study of family physicians' educational needs in the field of diabetes

**DOI:** 10.1186/1471-2296-12-27

**Published:** 2011-05-11

**Authors:** Suzanne Murray, Patrice Lazure, Sara Schroter, Philipp J Leuschner, Peter Posel, Thomas Kellner, Richard D Jenkins

**Affiliations:** 1 XDEV Group Inc., 210-8, Place du Commerce, Brossard, Quebec, Canada; 2 BMJ Editorial Office, BMJ Group, BMA House, Tavistock Square, London, UK; 3 EIMSED, Rotenturmstrasse 27/6, 1010 Vienna, Austria; 4 QUAIME, Seestrasse 7, CH-6454 Flüelen, Switzerland; 5 MSD, Richard Reitzner Allee 1, 85540 Haar, Germany; 6 BMJ OnExamination, Cardiff Medicentre, Heath Park, Cardiff, UK

## Abstract

**Background:**

The optimal care of persons with diabetes by general practitioners and family physicians (GP/FP) is complex and requires multiple competencies. This is a fairly unrecognized key challenge in the healthcare systems. In some cases, local and national Continuous Professional Development (CPD) initiatives target these challenges; however there have been few international initiatives, possibly because challenges emerging from different studies have not been linked across national boundaries. In this context, the authors have compiled data about gaps and/or barriers inherent to GP/FP care of persons with type 2 diabetes from Austria, Canada, Germany and the United Kingdom.

**Methods:**

Secondary analyzes of pre-existing studies were conducted to identify challenges in the care of patients with type 2 diabetes as faced by GPs/FPs. Two sources of data were reviewed: unpublished research data from collaborating organizations and articles from a literature search (in English and German). Articles retrieved were scanned by the research team for relevance to the study objectives and to extract existing gaps and barriers. The identified challenges were then categorized along three major axes: (1) phase of the continuum of care {from screening to management}; (2) learning domain {knowledge, skills, attitudes, behavior, context}; and (3) by country/region. Compilation and categorization were performed by qualitative researchers and discrepancies were resolved through discussion until concordance was achieved.

**Results and discussion:**

Thirteen challenges faced by GPs/FPs in the care for patients with type 2 diabetes were common in at least 3 of the 4 targeted countries/regions. These issues were found across the entire continuum of care and included: pathophysiology of diabetes, diagnostic criteria, treatment targets assessment, drugs' modes of action, decision-making in therapies, treatment guidelines, insulin therapy, adherence, management of complications, lifestyle changes, team integration, bureaucracy and third-party payers. The issues reported were not restricted to the physicians' knowledge, but also related to their skills, attitudes, behaviours and context.

**Conclusions:**

This study revealed challenges faced by GPs/FPs when caring for patients with diabetes, which were similar across international and health system borders. Common issues might be addressed more efficiently through international educational designs, adapted to each country's healthcare system, helping develop and maintain physicians' competencies.

## Background

An estimated 260 million people are affected by type 2 diabetes worldwide and a further 7 million people develop diabetes each year [1]. Serious consequences of this disease include a life span that may be shortened by 5 to 10 years. Additionally, diabetes will account for 6.8% of global mortality in the 20-79 age group for the year 2010 [1]. Furthermore, diabetes and its complications have been shown to have a significant effect on the quality of life (QoL), including reduced mobility, anxiety towards long-term consequences, and depression [2]. The importance of QoL has been recognized not only as an important outcome of diabetes care, but also as an important factor in adherence to diabetes self-care activities [3].

In addition, the cost of diabetes to the health care system is increasingly important. The International Diabetes Federation (IDF) [1] reports that most countries are predicted to spend between 5% and 13% of their total healthcare dollars on diabetes in 2010. The IDF expect the worldwide cost to treat and prevent diabetes and its complications to total at least 376 billion US$ in 2010, and to exceed 490 billion US$ by 2030. The IDF estimates that an average of 703 US$ per person will be spent on diabetes in 2010 globally.

The high prevalence/incidence of type 2 diabetes and its substantive effects on quality of life, social impact, and economic costs point to the importance of assuring that this disease is screened, diagnosed and managed expeditiously and effectively. Currently, the majority of persons with diabetes are diagnosed, treated, and managed by General Practitioners (GPs) and Family Physicians (FPs) as front line providers of care [4], rather than by a diabetologist or an endocrinologist [5]. Optimal care in type 2 diabetes requires practitioners to be competent in complexities of disease management as well as in patient communication, counseling, and education, as adressing patients' psychological problems and barriers are believed to improve diabetes oucomes [6]. FPs/GPs are therefore required to master both physiological and psychosocial approaches to the treatment and management of type 2 diabetes [6]; yet, research evidence shows that many are challenged in doing so [6,7].

A large study covering 13 countries, including Germany and the United Kingdom, found health care providers lacked resources (skill, time and referral sources) for commonly reported psychosocial problems amongst diabetes patients [6].

Additional studies have suggested that FPs/GPs experience challenges in treating and managing diabetes [8]. One study reported patients' frustration about their practitioners' lack of understanding of their perspective, as well as practitioners' frustration with patients' inability to achieve objectives and argued that frustration leads to decreased adherence and eventually, lower health outcomes [9]. In another study, physicians, despite acknowledging the psychosocial aspects of treatment adherence, focused almost entirely their management approach on blood glucose levels [10]. The same study linked adherence to treatment regimes with patient-provider communication [10]. Adherence is important as it has been estimated that 20% of patients had poor adherence (defined as taking incomplete medication at least 20% of the days) [11]. Another study identified physicians' lack of proactive intensification of therapy as an important barrier to optimal management of diabetes [12]. Less than optimal achievement of treatment and risk factors targets [13,14], and gaps in diagnosis and initial management of diabetes [15] have also been reported. There is also evidence indicating that FPs/GPs are challenged when intensification of medication is required [7,13]. However, the majority of these issues were studied solely in the context of a single country.

Continuing Professional Development (CPD) programs are fundamental to the development and maintenance of physician competencies [16] and have been developed and deployed locally to address the aforementioned challenges. Cultural differences and different health system contexts are likely to influence the precise nature of clinical gaps and barriers in a given country, however it is likely that similar challenges will arise in different countries. Identifying the issues that are concurrently rising across borders would allow CME providers to deploy and coordinate international CME interventions. Through international deployment, scarce resources can be shared, and programs developed more efficiently.

The organizations represented by the authors of this manuscript formed an international collaborative with the goal of developing and disseminating international educational initiatives to FPs and GPs, and of addressing gaps and challenges in the care of persons with diabetes. The collaborative regroups organizations from Canada, Austria, Germany, and the United Kingdom. The overall objective of this research, which represents the first step of the international collaborative project, was to identify similarities in the gaps and barriers of the four regions that could be used to develop an international educational program. In order to achieve that, the following two steps were undertaken:: firstly, international data characterizing gaps and/or barriers inherent to FPs and GPs care of persons with type 2 diabetes was compiled; and secondly, the identified gaps and/or barriers were categorized according to the phases in the continuum of care (screening, diagnosis, treatment, management, referral) and the adapted domains of Bloom's taxonomy of learning [17] (knowledge, skills, attitudes, behavior, or context).

## Methods

### Data collection

This article presents secondary post-hoc analyzes of pre-existing studies, from two sources: literature reviews (in English and German) and unpublished research data from the co-authors' organizations. The three studies of the co-authors were developped and deployed independently, and each used its own methodology, described later in this section.

### Literature Review

The English-language literature review included three databases: Medline [18], EMBase [19] and the Education Resources Information Centre (ERIC) [20]. The following search keywords and logic were adapted and complemented when necessary for each database: Diabetes AND (Gaps OR Barriers OR Needs Assessment OR Health Care Needs OR Challenges) AND (Family Practice OR Family Medicine OR General Practice). Search keywords were not restricted to any particular field. Article abstracts were screened by the research team for relevance to the study objectives.

The German-language literature review included the following literature databases: Medline [18], EMBase [19], the *Deutsches Institut für Medizinische Dokumentation und Information *(DIMDI) [21], the *Zeitschriftendatenbank *(ZDB) [22] and the Current Contents *Medizin *(ccMed) Database [23]. The following search keywords and logic were adapted and complemented when necessary for each database: Diabetes AND (Gaps OR Barriers OR Needs Assessment OR Health Care Needs OR Challenges OR *Versorgungsforschung*) AND (*Hausarzt *OR *Arzt für Allgemeinmedizin *OR *Praktischer *Arzt OR *Allgemeinmedizin*). Search keywords were not restricted to any particular field. Article abstracts were scanned by the German research team for relevance to the study objectives.

The search to identify gaps and barriers was limited to articles published between 2007 and 2010 inclusively. Articles were included if they reported gaps, barriers or challenges encountered by FPs/GPs in the care of type 2 diabetes persons, but were not limited to needs assessments *per se*, nor were they limited by the methodology used. Articles had to report on gaps and barriers in at least one of the four geographical areas selected (Austria, Canada, Germany and United Kingdom). Final selection of the articles was performed by the co-authors and research assistants. No formal quality appraisal of each methodology was performed.

### Definitions used

In CPD, a gap is defined as "*the difference between the present condition of the learner and the acceptable norm*" [24]. Barriers are defined as "*tangible or intangible obstructions that block, hinder, delay, deter, or interfere with action, movement, progress, or development*" [25]. These definitions are widely recognized and have become a standard in the CPD field.

### Research data from co-authors

The European Institute for Medical and Scientific Education's Diabetes Needs Assessment (EIMSED-NA) consisted of a detailed questionnaire covering the phases from early diagnosis to management in the continuum of care of type 2 diabetes. In Austria, Germany, and the United Kingdom, the questionnaire was applied to a sample number of 100 GPs in each country via computer assisted telephone interviews (25-30 minutes per interview) during February 2009. Participants were purposively selected to achieve a spread of the interviewees over the respective countries. The results were statistically analysed and graphically displayed for subsequent scientific review.

Secondary analysis of the BMJ's Diabetes Needs Assessment Tool (DNAT) Trial [26] was conducted by onExamination, part of BMJ Group. Quantitative assessment of knowledge gaps and qualitative analysis of actual changes to practice as a result of the learning intervention was conducted in a sample of 677 English and German speaking physicians recruited for the online learning research study in the field of diabetes (BMJ-GAP). Data was collected during 2009. The responses to the test-items within the DNAT were analysed and those that highlighted the most prominent knowledge gaps identified. The subject matter of the test-items were classified and grouped into common areas representing gaps in knowledge. The initial data analysis of the DNAT trial was conducted by independent statisticians and is reported elsewhere [26].

The AXDEV Diabetes Needs Assessment (AXDEV-NA) used a mixed-methods approach, combining both qualitative (discussion groups) and quantitative (questionnaire) data collection techniques to identify educational opportunities for FPs/GPs in type 2 diabetes care [27]. The study used a triangulated research design, where multiple data collection methods are combined to examine the same phenomena from different perspectives, strengthening the trustworthiness and validity of the evidence [28,29]. The study was conducted in 2008 and included 22 Physicians, 13 allied health professionals and 8 patients and covered the whole continuum of care, from screening and testing, to treatment and management.

### Analysis

Articles identified by the literature review, as well as internal reports from the EIMSED-NA, BMJ-GAP and AXDEV-NA studies were reviewed by the interdisciplinary research team to extract the gaps and barriers, as well as the country(ies) in which the challenges were observed. Extraction was performed based on the definitions of gaps and barriers provided above. The issues listed were then compiled and closely related ones were regrouped together, by experienced qualitative researchers, including co-authors PL and SM. Grouping was performed by idenfication of direct synonyms, shared terminology and/or evidence of a higher level theme.

Common gaps and barriers, i.e., observed in at least three of the four countries, were selected and coded according to the phase in the continuum of care (screening, diagnosis, treatment, management, referral, context) and to whether the gap and/or barrier existed in physicians' knowledge, skills, attitudes, behavior, or in context, as described in the original study(ies). In the continuum of care categorization, context related refers to issues that are not related to a phase of care in particular but may affect any of the phases, while context related in the second classification refers to issues that are more systemic and not at the individual level of the physician, such as re-imbursement policies for example. Coders were experienced qualitative researchers, including co-authors PL and SM. Discrepancies throughout the process were resolved through discussion until concordance was achieved.

## Results

### Literature review

The search strategy in the English-language literature review produced 136 references in Medline, 148 in EmBase and 22 in ERIC, while the German-language literature review produced 72 references in Medline, 84 in EmBase, 97 in DIMDI and 33 in ccmed. After the elimination of duplicates and the application of our research criteria, 16 documents in English and 22 in German, in which FPs/GPs from the identified countries faced gaps or barriers providing optimal care of type 2 diabetes, were retained for further evaluation.

### Data extraction, compilation and classification

A total of 98 gaps and barriers were extracted from the 38 articles found in the English and German literature reviews and the three studies from the co-authors. Similar issues were then grouped to produce a list of 31 gaps and barriers, 13 of which were common in at least 3 of the 4 targeted countries. Table 1 shows the classification of these 31 gaps and barriers, highlighting the 13 that were retained.

**Table 1:** Classification of the gaps/barriers identified in the targeted countries/region.

		Country/Region				Type					
**Phase of the Continuum of Care**	**Themes: Gaps and barriers**	**Austria**	**Canada**	**Germany**	**UK**	**Knowledge**	**Skill**	**Attitude**	**Behaviour**	**Context**	**References**

Condition	**Pathophysiology of diabetes**	X		X	X	X					EIMSED-NA

Diagnosis	**Diagnosis criteria**		X	X	X		X	X			AXDEV-NA, BMJ-GAP, [47,48]
	
	Pre-diabetes			X	X		X				BMJ-GAP, [43]
	
	Lack of screening		X					X	X	X	AXDEV-NA

Testing	**Assessment of treatment targets**	X	X	X	X		X	X			AXDEV-NA, BMJ-GAP, [30-32,40,49,50]

Treatment	**Explaining drugs' modes of action **	X		X	X	X	X				BMJ-GAP, EIMSED-NA, [48]
	
	**Decision-making in therapies**	X	X	X	X		X	X	X		AXDEV-NA, BMJ-GAP, EIMSED-NA, [48,51-54]
	
	**Treatment guidelines**	X		X	X	X					EIMSED-NA, [34,35,55]
	
	**Insulin therapy**	X		X	X	X	X	X			BMJ-GAP, EIMSED-NA, [36,37,56]

Management	**Adherence**	X	X	X	X		X	X	X	X	AXDEV-NA, EIMSED-NA, [38,39]
	
	**Management of complications**	X		X	X	X	X	X	X		BMJ-GAP, EIMSED-NA, [41,57-59]
	
	**Lifestyle changes**		X	X	X		X	X			AXDEV-NA, BMJ-GAP, [43,44,60]
	
	Patient education		X	X		X	X				AXDEV-NA-NA, BMJ-GAP-GAP, [44]
	
	Blood pressure control		X						X		[61]
	
	Tele-monitoring		X					X		X	[62]
	
	Computerization of management		X					X	X	X	[2]
	
	Psychological care		X		X	X	X	X	X		AXDEV-NA, [63]
	
	Motivation for long-term follow-ups		X				X	X	X		AXDEV-NA
	
	Interprofessional teamwork		X			X		X			[48]
	
	Lack of secondary support				X					X	[64]

Referral	**Suboptimal team integration**		X	X	X		X	X	X		BMJ-GAP, [45,47,48]

Context	**Bureaucracy**	X	X	X	X				X	X	AXDEV-NA, EIMSED-NA
	
	Long waiting lists		X							X	[65]
	
	Care for the aboriginal population		X							X	[40]
	
	Access and cost		X							X	[40,65,66]
	
	**Third party payers**	X		X	X					X	EIMSED-NA, [67]

Other	Physicians' resistance to change				X			X	X		[54]
	
	Lack of resources				X					X	[54]
	
	Physicians' bias towards obesity		X					X			[68]
	
	Disengagement of patients		X				X	X			AXDEV-NA
	
	Provider-Patient relationships		X				X	X	X		AXDEV-NA

Note: Gaps and barriers presented in bold are those that were common in at least 3 of the 4 targeted countries.

### Gaps and barriers: Pathophysiology of Diabetes

Concerning the condition of diabetes, the EIMSED-NA study revealed that FPs/GPs lacked exact knowledge of the pathophysiology of diabetes. More precisely, the pathophysiology section of the study had questions on the topic of pro-insulin, as well as on the effect of diabetes on the pancreatic hormones, beta cell mass and adiponectin. This was observed in the three regions covered by the EIMSED-NA study, i.e., Austria, Germany and the UK.

### Gaps and barriers: Diagnosis

Challenges in diagnosing diabetes were identified in the UK [BMJ-GAP], Germany [BMJ-GAP] [EIMSED-NA], and Canada [AXDEV-NA]. It was found that FPs/GPs lacked exact knowledge of the diagnostic criteria and cut-off points. According to the AXDEV-NA study, diagnosis guidelines are perceived to change often because of frequent updates, leading each Canadian health care organization to develop their own cut-off points. This result shows that the application of diagnosis guidelines can not be related solely to knowledge, but that attitudes have an impact as well.

### Gaps and barriers: Testing

It was found that treatment targets for risk factors (HbA1c, blood pressure, LDL-C) were not being adequately tested in the UK and Germany [BMJ-GAP, EIMSED-NA], in Austria [EIMSED-NA], as well as in Canada [AXDEV-NA]. In Canada, physicians overestimate the level at which the targets are achieved, a tendency that, when combined with the perception that such testing is expensive, impractical and time consuming, leads FPs/GPs to test less regularly [AXDEV-NA]. Studies report that in Germany, HbA1c is tested in only 68% of patients [30]. Furthermore, only 7.6% of diabetic persons reached target blood pressure values of < 130/80 mm Hg, and target LDL-C < 100 mg/dL (< 2.6 mmol/L) was achieved in only 15.8% of patients [31]. In Germany and the UK it was reported that physicians lacked the skills required to educate patients how to monitor their glucose levels [BMJ-GAP].

### Gaps and barriers: Treatment

In Austria, Germany and the United Kingdom, it was reported that FPs/GPs lack the exact knowledge of the mode or mechanism of action of the different drugs they prescribe [EIMSED-NA]. Additionally, the BMJ-GAP analysis showed, for the UK and Germany, that FPs/GPs lack the ability to give clear explanations to the patients of how the substances work.

When treating persons with type 2 diabetes, FPs/GPs in all four targeted countries lacked the knowledge, skills and attitude to optimize their decision-making related to therapies [AXDEV-NA, EIMSED-NA]. Notably, FPs/GPs lacked knowledge about new treatment strategies recently added to guidelines [32], and did not feel competent in the correct and safe use of newly developed therapies. They also lacked the confidence to prescribe new treatments with which they are unfamiliar. Furthermore, in the three targeted European countries, FPs/GPs lacked knowledge of the guidelines for treating diabetes, and of the recommendations of the diabetes associations [EIMSED-NA]. Guidelines were perceived as contradictory, leading physicians to rely on treatment strategies with which they are familiar [EIMSED-NA]. In Germany, treatment guidelines were viewed with skepticism by FPs/GPs, describing them as disregarding the individual patient's reality, especially in the case of patients with multiple morbidities [33,34].

The use of insulin therapy was expressed as a challenge for FPs/GPs in the three European countries [EIMSED-NA]. More specifically, FPs/GPs did not feel competent with insulin therapy, lacked knowledge on when to initiate insulin therapy, and had challenges in maintaining and adjusting insulin therapy [BMJ-GAP]. In Germany, they were challenged in initiating an insulin therapy after failure of the treatment with oral anti-diabetics [35] and they lacked knowledge and skills regarding the appropriate and individualized treatment of the elderly (largely due to specificity in the guidelines regarding this group) [36].

### Gaps and barriers: Management and Monitoring

In the management and monitoring of type 2 diabetes, FPs/GPs from all four countries were found to lack formal training in counseling and coaching the patient [AXDEV-NA] [EIMSED-NA] [37], a gap which has been shown in the literature to impact on patient motivation and adherence [9]. FPs/GPs had developed suboptimal therapeutic relationships and they lacked the skills to actively engage their patients in their health management. They had little understanding of the psycho-social issues underlying adherence, and appropriate tools to assess potential for and actual adherence of their patients is lacking [27]. In Germany, a lack in shared decision making was reported [38]. Context issues may also play a role in adherence as 57% of surveyed Canadians living with diabetes said they do not comply with their prescribed therapy due to cost and lack of access [39].

FPs/GPs from the three European countries found it challenging to manage the complications of diabetes on a long-term basis particularly neuropathies, pancreas pathologies, diabetic foot [BMJ-GAP, EIMSED-NA], and retinopathy [40]. In Germany [41], challenges in skills, knowledge and attitudes were reported related to optimal risk-assessment of the complications that a patient might develop within a ten-year period. More specifically, there was a large disparity among the FPs/GPs in their approach to risk-assessments, because FPs/GPs were more inclined to go with their "gut feeling" than making a decision based on knowledge, competence and/or skills.

Results [AXDEV-NA, BMJ-GAP, [40,42]] also indicated that British, Canadian and German FPs/GPs are not being equipped to provide the appropriate patient education to support patients in their efforts towards lifestyle changes which would improve their condition. In Germany, deficits were found in FP/GPs' knowledge/skill/attitude to motivate the patients for healthy lifestyle changes [37]. Furthermore, in Canada [AXDEV-NA] and Germany [43], it has been shown that FPs/GPs often put their focus on curative care, thus reducing the emphasis on and neglecting prevention.

### Gaps and barriers: Referral

Suboptimal integration of the diabetic team during the referral process was also reported in the United Kingdom, Canada and Germany. More precisely, a gap was found in the United Kingdom and Germany, relating to the suboptimal use of the diabetic team, especially in referral to other team members, e.g., specialist diabetic nurse, podiatrist and dietician [BMJ-GAP]. In Germany, a lack of resources and support for diabetic teams was reported as a barrier, even though the potential of the paramedical personnel is high, which could mean that the resources are there, but underutilized [44,45]. In Canada, suboptimal integration between healthcare professionals was observed [AXDEV-NA], as well as a lack of knowledge of other professionals' contributions [46].

### Gaps and barriers: Context Issues

Context-related issues were also identified in all four countries; more specifically, bureaucracy was named as a barrier to optimal care. The high level of documentation and effort required to update records were perceived as problematic [EIMSED-NA]. Furthermore, FPs/GPs in diabetes care play many roles for which they were not formally trained, including carrying out administrative tasks [AXDEV-NA].

Additionally, third party payers were identified as context-related issues in Austria, Germany and the United Kingdom [EIMSED-NA]. Limitations set by health insurance bodies/cost carriers on the prescribing of newer (and in most cases, more expensive) drugs are perceived as a barrier to optimal treatment by physicians [EIMSED-NA].

## Discussion

The consensus approach used for this research identified needs, gaps and challenges that are similar in four different countries/regions. Issues identified as common to the targeted countries/regions might also be found in other countries, but further research is required to confirm this. These issues represent potential barriers to optimal healthcare that could be addressed by international educational design. Although the international collaborative has limited its efforts to four countries/regions in its first phase of activity, subsequent phases will be gradually enlarged to include additional countries, and assess the feasibility of using the same approach at a larger and more global level.

Education researchers designing needs assessments in diabetes in these and other countries should be aware of the most common gaps and barriers presented here, and assure that they are included in future research designs.

International educational design would potentially enable important economies of scale for education providers. The potential economies related to CME internationalization could be very important in optimizing resource utilization in healthcare performance improvement, therefore evolving the applied standards in the CME field. Reaching those highest standards is required to help physicians and other health care providers achieve patient outcomes compliant with the current best evidence, while respecting existing healthcare budgets.

Cultural differences and different health system contexts will require tailoring to ensure international applicability. Nonetheless, education providers and designers should verify the comparability of educational activities and look for similarities that could be included in internationally deployed programs. Reviews such as this one could be instrumental in tailoring programs to local and regional needs.

The approach presented here could also be used to identify common gaps across countries in other therapeutic areas where internationalization of CPD activities could also be beneficial.

### Limitations

We acknowledge that our approach has some limitations. Firstly, only recent manuscripts indexed in Medline, EmBase, ERIC, DIMDI, ZDB or ccMED were included, providing a selected sample of the literature. To supplement our review, we drew upon three relevant sources of data known to us. Secondly, needs, gaps and barriers that were not identified in at least three of the four targeted countries/regions were excluded from further analysis/discussion and some of these issues could still hold international value. However our approach was designed to identify issues clearly relevant to multiple countries. Needs, gaps and barriers identified in less than three of the targeted countries/regions, require more exploration to determine if they are unique to one or two countries or if they are international issues that have not yet been identified in the other targeted countries. Thirdly, it cannot be assumed that care is optimal in other areas where gaps and barriers were not identified because emphasis was put on detecting common challenges, at the expense of areas in which care is excellent. Finally, cultural differences and different health system contexts might limit the generalisability of the findings so these issues should be investigated before designing local educational interventions in other areas.

### Next steps

Following the literature-based needs assessment presented in this manuscript, the international collaborative initiative is pursuing its objective of internationalizing CME activities in the field of Diabetes, as it extends its efforts to additional countries. An international educational program is being developed to address five of the gaps and barriers revealed by this review. This activity will focus on diagnostic criteria, suboptimal integration of diabetic teams, assessment of treatment targets, modes of action of drugs and treatment guidelines. Those five gaps were selected during a face-to-face meeting with all members of the international collaborative, where members discussed and ranked each of the 13 gaps identified according to their reported importance and impact on patients' outcomes. Evaluation of the international educational program, including the selection process of the five gaps to be targeted, will be submitted for a separate publication.

## Conclusions

A consensus approach identified needs, gaps and challenges faced by GPs/FPs when caring for persons with type 2 diabetes that are similar in Austria, Canada, Germany and the United Kingdom. These issues represent potential barriers to optimal healthcare that could be addressed by international educational design. CPD internationalization could quickly evolve the applied standards in medical education, leading to the optimization of resource utilization in healthcare performance improvement. Elevation of the CPD standards will ultimately elevate the physicians' performance in providing care to their diabetes patients, and consequently contribute to better patient outcomes.

## Competing interests

SM is president and founder of AXDEV Group Inc., Brossard, Canada. PL is a researcher at AXDEV Group Inc., Brossard, Canada. SS is employed by BMJ Group which provides online learning resources for health professionals and regularly undertakes research on the behalf of BMJ Group. PJL is a Medical Scientific Liaison Manager at EIMSED, Vienna, Austria. PP is CEO at QUAIME AG in Flüelen, Switzerland. TK is leader of the Global Medical Education Strategy at Merck, New Jersey, United States. RDJ is a medical education advisor to onExamination and Editor-in-chief of BMJ Case Reports.

## Authors' contributions

SM conducted the AXDEV Diabetes Needs Assessment [AXDEV-NA] and presented the findings to the other members of the collaborative. SM and PL carried out the English literature review, compiled and classified the gaps, and wrote the first draft of this manuscript. SS and RDJ conducted the gap analysis of the data from the DNAT trial [BMJ-GAP] and presented the findings to the other members of the collaborative. PJL conducted the EIMSED needs assessment [EIMSED-NA] and presented the findings to the other members of the collaborative. PP carried out the German literature review. All authors contributed to the final analysis of common gaps, critically revised the first draft of the manuscript, and read and approved the final version of the manuscript.

## Pre-publication history

The pre-publication history for this paper can be accessed here:

http://www.biomedcentral.com/1471-2296/12/27/prepub
